# Recent Developments in DFTB+, a Software Package for Efficient Atomistic Quantum Mechanical Simulations

**DOI:** 10.1021/acs.jpca.5c01146

**Published:** 2025-06-06

**Authors:** B. Hourahine, M. Berdakin, J. A. Bich, F. P. Bonafé, C. Camacho, Q. Cui, M. Y. Deshaye, G. Díaz Mirón, S. Ehlert, M. Elstner, T. Frauenheim, N. Goldman, R. A. González León, T. van der Heide, S. Irle, T. Kowalczyk, T. Kubař, I. S. Lee, C. R. Lien-Medrano, A. Maryewski, T. Melson, S. K. Min, T. Niehaus, A. M. N. Niklasson, A. Pecchia, K. Reuter, C. G. Sánchez, C. Scheurer, M. A. Sentef, P. V. Stishenko, V. Q. Vuong, B. Aradi

**Affiliations:** † SUPA, Department of Physics, 3527 University of Strathclyde, John Anderson Building, 107 Rottenrow, Glasgow G4 0NG, U.K.; ‡ Consejo Nacional de Investigaciones Científicas y Técnicas (CONICET), Instituto de Investigaciones en Fisicoquímica de Córdoba (INFIQC), X5000HUA Córdoba, Argentina; § Universidad Nacional de Córdoba, Facultad de Ciencias Químicas, Departamento de Química Teórica y Computacional, X5000HUA Córdoba, Argentina; ∥ Universidad Nacional de Córdoba, Centro Láser de Ciencias Moleculares, X5000HUA Córdoba, Argentina; ⊥ Bremen Center for Computational Materials Science, 9168 University of Bremen, Bremen 28359, Germany; # Max Planck Institute for the Structure and Dynamics of Matter, Center for Free-Electron Laser Science, Luruper Chaussee 149, Hamburg 22761, Germany; ¶ School of Chemistry, University of Costa Rica, San José 11501-2060, Costa Rica; ∇ Department of Chemistry, 1846 Boston University, Boston, Massachusetts 02215, United States; ○ Department of Chemistry, Advanced Materials Science & Engineering Center, and Institute for Energy Studies, 1632 Western Washington University, Bellingham, Washington 98225, United States; ⧫ Condensed Matter and Statistical Physics, The Abdus Salam International Centre for Theoretical Physics, Trieste 34151, Italy; †† Microsoft Research, AI for Science, Schipol 1118 CZ, The Netherlands; ‡‡ Institute of Physical Chemistry (IPC), 150232 Karlsruhe Institute of Technology, Karlsruhe 76131, Germany; §§ School of Science, Constructor University, Bremen 28759, Germany; ∥∥ 4578 Lawrence Livermore National Laboratory, Livermore, California 94550, United States; ⊥⊥ Department of Chemical Engineering, University of California, Davis, California 95616, United States; ## Computational Sciences and Engineering Division, 6146 Oak Ridge National Laboratory, Oak Ridge, Tennessee 37831, United States; ¶¶ Center for Multidimensional Carbon Materials (CMCM), Institute for Basic Science (IBS), Ulsan 44919, Republic of Korea; ∇∇ 531612 Max Planck Computing and Data Facility, Garching 85748, Germany; ○○ Department of Chemistry, Ulsan National Institute of Science and Technology (UNIST), 50 UNIST-gil, Ulju-gun, Ulsan 44919, Republic of Korea; ⧫⧫ Univ Lyon, Université Claude Bernard Lyon 1, CNRS, Institut Lumière Matière, Villeurbanne F-69622, France; ††† Theoretical Division, 5112 Los Alamos National Laboratory, Los Alamos, New Mexico 87545, United States; ‡‡‡ Institute for the Study of Nanostructured Materials, CNR, Roma 00010, Italy; §§§ Fritz Haber Institute, Berlin 14195, Germany; ∥∥∥ Instituto Interdisciplinario de Ciencias Básicas (ICB-CONICET), Universidad Nacional de Cuyo, Mendoza 5502, Argentina; ⊥⊥⊥ Institute for Theoretical Physics, 9168 University of Bremen, Bremen 28359, Germany; ### Max Planck Institute for the Structure and Dynamics of Matter, Center for Free-Electron Laser Science (CFEL), Luruper Chaussee 149, Hamburg 22761, Germany; ¶¶¶ Cardiff Catalysis Institute, School of Chemistry, Cardiff University, Park Place, Cardiff, Wales CF10 3AT, U.K.

## Abstract

DFTB+ is a flexible, open-source software package developed by its community, designed for fast and efficient atomistic quantum mechanical simulations. It employs various methods that approximate density functional theory (DFT), such as density functional-based tight binding (DFTB) and the extended tight binding (xTB) approach allowing simulations of large systems over extended time scales with reasonable accuracy, while being significantly faster than traditional ab initio methods. In recent years, several new extensions of the DFTB method have been developed and implemented in the DFTB+ program package in order to improve the accuracy and generality of the available simulation results. In this paper, we review those enhancements, show several use case examples and discuss the strengths and limitations of its features.

## Graphical abstract

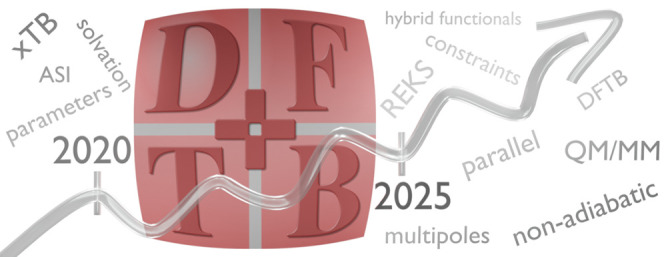

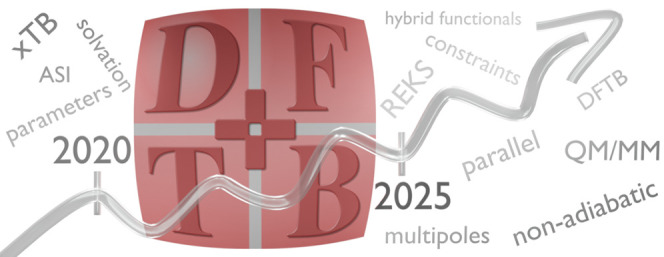

## 1 Introduction

Density functional theory (DFT) is a widely used electronic structure method known for its balance of accuracy and computational efficiency, making it the preferred choice for modeling large, chemically complex systems. However, for even larger systems and longer time scales, force-field models often take precedence in materials and chemical simulations. Bridging the gap between these two approaches are semiempirical methods, which rely on approximations to Hartree–Fock or DFT. Among these, the family of density functional tight binding (DFTB) methods, providing a simplified version of DFT by reducing the complexity of Kohn–Sham DFT into a tight binding framework.

This paper highlights the recent developments of DFTB+, an open-source software package designed to consolidate this family of methods and make them accessible to the chemical, materials, and condensed matter research communities. The article summarizes the key features of the DFTB method, and presents the latest developments in the method as well as in the DFTB+ code since its initial release in 2007,^1^ with particular focus on developments made since the previous in-depth description of the code in 2020.^2^

## 2 Self-Consistent Tight Binding Derived from DFT

DFTB and xTB represent a family of models derived from Kohn–Sham DFT (KS-DFT) through an expansion of the total energy functional. In this approach, the ground state density ρ­(**r**) = ρ_0_(**r**) + δρ­(**r**) is expressed as a fluctuation δρ around a suitably chosen reference density ρ_0_. This reference density is typically constructed as superposition of the neutral electron densities, ρ_*A*_, of the constituent atoms {*A*}. The total energy functional is then expanded up to third order in these density fluctuations

1 $$E\left[\rho_{0}+\delta \rho \right]\approx E^{0}\left[\rho_{0}\right]+E^{1}\left[\rho_{0},\delta \rho \right]+E^{2}\left[\rho_{0},{\left(\delta \rho \right)}^{2}\right]+E^{3}\left[\rho_{0},{\left(\delta \rho \right)}^{3}\right]$$

The inclusion and approximation of the terms in this expansion define the different levels of the DFTB method: DFTB1, DFTB2 and DFTB3,^2–6^ as well as the xTB models, GFN0-xTB, GFN1-xTB and GFN2-xTB.^7–10^ Both DFTB and xTB share a similar derivation and overall model structure; the primary distinction lies in how their parameters are derived and treated.

### 2.1 Density Functional Tight Binding

#### 2.1.1 DFTB1

The DFTB1 method approximates the first two terms in eq 1 by employing the following strategies: (i) the Kohn–Sham orbitals Ψ_*i*_(**r**) are expressed using a minimal basis set of confined atomic orbitals ϕ_μ_(**r**) and unknown coefficients *c*_*i*μ_: Ψ_*i*_(**r**) = ∑_μ_ *c*_*i*μ_ϕ_μ_(**r**). These atomic orbitals are computed by solving atomic Kohn–Sham equations under an additional confining potential, which slightly compresses them with respect to the free atomic wave functions. This enhances the representation of the electron density in the chemically active binding regions. The DFTB1 Hamiltonian matrix elements $H_{\mu \nu }^{0}=\left\langle \phi_{\mu }\left|Ĥ\left[\rho_{0}\right]\right|\phi_{\nu }\right\rangle$ are then explicitly calculated and tabulated using a two-center approximation.^11^ (ii) The energy contribution *E* ^0^[ρ_0_] is represented by fast decaying two-body repulsive potentials, *V*_*AB*_^rep^, with a cutoff typically between first and second neighbor distances. The DFTB1 total energy then reads

2 $$E^{DFTB1}=\sum_{i}\sum_{AB}\sum_{\mu \epsilon A,\nu \epsilon B}c_{i\mu }^{*}c_{i\nu }H_{\mu \nu }^{0}+\frac{1}{2}\sum_{AB}V_{AB}^{rep}$$

Although *H*_μν_^0^ only depends on the reference density and represents, therefore, a zero-order term, the total energy *E* ^DFTB1^ is a first order term in the density fluctuations due to the product of the atomic orbital coefficients *c*_*i*μ_^*^ *c*_*i*ν_. The parametrization of DFTB1 requires the construction of integral tables for $\left\langle \phi_{\mu }\left|Ĥ\left[\rho_{0}\right]\right|\phi_{\nu }\right\rangle$ and $\left\langle \phi_{\mu }|\phi_{\nu }\right\rangle$ , evaluated at specific interatomic distances and orientations for all relevant basis function combinations. These precomputed tables enable the rapid assembly of the Hamiltonian and overlap matrices (*H*_μν_^0^ and *S*_μν_) for a given geometry using efficient Slater–Koster transformations^12^ during the calculation.

#### 2.1.2 DFTB2 + DFTB3

DFTB1 is a so-called non-self-consistent electronic structure method, because determining the coefficients *c*_*i*μ_ and evaluating *E* ^DFTB1^ require only a single diagonalization of the Hamiltonian matrix. Extending the method to include the *E* ^2^ and *E* ^3^ terms leads to a simple, but self-consistent computational scheme, if the charge density fluctuations are approximated as spherical atomic charge densities. Under this approximation, the relevant integrals can be computed analytically and are represented by two-body functions γ_*AB*_, which describe the interaction between atomic point charges Δ*q*_*A*_. The total energy for DFTB3 is given by

3 $$E^{DFTB3}=E^{DFTB1}+\frac{1}{2}\sum_{AB}\Delta q_{A}\Delta q_{B}\gamma_{AB}+\frac{1}{3}\sum_{AB}\Delta q_{A}^{2}\Delta q_{B}\Gamma_{AB}$$

where Δ*q*_*A*_ values are typically computed using a Mulliken charge scheme. DFTB2 includes only the first two terms and is suitable for systems where Δ*q*_*A*_ are sufficiently small, such as most neutral organic or inorganic materials. DFTB3 also incorporates the third-order term involving Γ_*AB*_, which is the derivative of γ_*AB*_ with respect to atomic charges. This term is crucial for small and charged molecules or systems where a charge is localized. The derivation of the analytical function γ_*AB*_ assumes a linear relationship between chemical hardness and atom size, which does not hold well for first row elements interacting with hydrogen. To address this, DFTB3 employs a modified γ^*h*^ function to account for these deviations empirically. Additionally, certain interactions, such as P–O or N–H, require specialized parameter sets. The primary limitations of DFTB1–DFTB3 arise from the integral approximations, the (confined) minimal basis set, the choice of the DFT functional employed in constructing ${Ĥ}^{0}$ ,^2^ and the monopole approximation for charge fluctuations. Some of these are addressed through recent extensions, including higher multipole orders (Section 3), and hybrid functionals (Section 5).

### 2.2 xTB

The GFN*n*-xTB methods (*n* = 0, 1, 2) use the same formal derivation starting from KS-DFT, but take an alternative strategy in several aspects.^9^ By formulating the total energy primarily in terms of one-center terms and employing an empirical Hamiltonian, these methods enable an easier parametrization which allowed a rapid parameter development across the periodic table up to *Z* = 86 already in the first publication.^7^ These models have been designed as special purpose tools,^9^ focusing the parametrization effort on geometries (G), vibrational frequencies (F) and noncovalent interactions (N), motivating the acronym GFN (the “x” refers to extensions in the AO basis set and the extended form of the Hamiltonian). DFTB+ implements the GFN1-xTB and GFN2-xTB methods (but not GFN0-xTB) by interfacing the open source tblite library.^10^

#### 2.2.1 GFN0-xTB

This is the lowest level approximation to eq 1 in the xTB method family. The repulsive energy term is expressed as

4 $$E_{rep}=\frac{1}{2}\sum_{AB}\frac{Z_{A}^{eff}Z_{B}^{eff}}{R_{AB}}e^{-\sqrt{\alpha_{A}\alpha_{B}}{\left(R_{AB}\right)}^{k_{f}}}$$

where the interaction depends on pairwise atomic distances *R*_*AB*_, element specific fitted parameters *Z*_*A*_^eff^ and α_*A*_, and a global constant *k*_f_. As an additional semiclassical component, the model also incorporates a total energy correction for the dispersion interaction via the D4 model.^13^ Furthermore, it contains a short-range correction term to improve covalent bonds, as well as an isotropic electrostatic energy term calculated by an electronegativity equilibration model.

The Hamiltonian-matrix is written in an extended Hückel-like fashion as

5 Hμν=12KABll′Sμν(Hμμ+Hνν)χ(ENA,ENB)Π(RAB,l,l′)ϒ(ζlA,ζl′B)⁣μ∈l(A),ν∈l′(B)

where $K_{AB}^{ll^{\prime }}$ are shell-specific (and in some cases element-pair-specific) scaling parameters. *H*_μμ_ and *H*_νν_ are the diagonal elements (influenced by the chemical environment through an empirical relation combining shell and element specific parameters *h*_*A*_^*l*^ with the atom’s coordination number). The quantity χ represents an electronegativity-dependent scaling factor, while Π is a polynomial scaling function of the distance, *R*_*AB*_, with element and shell specific fit parameters. Finally, ϒ is a correction term depending on the Slater-type atomic orbital exponents ζ_*A*_. In GFN0 and GFN1, it is set to one, while in GFN2 it aims to empirically incorporate effects analogous to kinetic energy functionals in ab initio theories. For further details on the individual terms in eq 5, and on the GFN-xTB methods in general, please refer to ref 9.

#### 2.2.2 GFN1-xTB

The GFN1-xTB method has similarities with the self-consistent DFTB3.^7^ It includes both second and third order terms from the beginning, for the latter, however, only the diagonal terms, which is sufficient for most applications.^6,14^ A different version of γ is applied to describe the electrostatics; the Mataga–Nishimoto–Ohno–Klopman formula is well-known from the traditional quantum chemical semiempirical methods. The functional dependence of both is, however, very similar.

#### 2.2.3 GFN2-xTB

At this level, adjustments in the first order terms are introduced, the main innovation going beyond GFN1-xTB and DFTB3 is the introduction of anisotropic second order terms and self-consistent dispersion treatment via the D4 dispersion model.^8^ This improves the description of, e.g., conformational energies and noncovalent interactions notably, and makes the method more transferable so that it allows, for example, to drop the specific halogen bonding term or the double-ζ description of hydrogen as introduced at the levels before.

#### 2.2.4 Spin–Orbit Coupling with xTB

The atomic spin–orbit constants for H to Og are available^15^ and can readily be applied for the GFN1-xTB and GFN2-xTB models using the existing DFTB+ infrastructure for two-component Hamiltonians.^16^

## 3 Multipole Expansion of the Coulomb Charges

In traditional DFTB2 and DFTB3 models, the charge fluctuations are represented using atom-centered monopole charges. However, the spherical symmetry of these atomic charge fluctuations impose limitations on accurately describing electrostatics for systems with highly anisotropic charge distributions. Bodrog and Aradi proposed a multipolar expansion model for the charge fluctuations up to dipolar terms,^17^ which was implemented for carbon-only systems.^18^ A similar approach was later adopted in the GFN2-xTB model,^8^ which extended the scheme to include monopole–quadrupole interactions. More recently, Vuong and co-workers presented a revised and extended version of the multipolar model for DFTB (mDFTB),^19^ that accounted for all interactions among monopole, dipole, and quadrupole moments.

This model is based on the DFTB2 framework and approximates the second order kernel (the sum of Coulomb and exchange–correlation kernels) using the function γ­(**r**, **r**′), which is subsequently expanded into a Taylor series around atomic sites. Considering interactions among the first three orders of the charge fluctuation, the energy expression for the mDFTB2 model is written as

6 EmDFTB2=EDFTB1+12∑A,B[ΔqAfAB(00)ΔqB+ΔdA·fAB(11)·ΔdB+ΔQA·fAB(22)·ΔQB+2ΔqAfAB(01)·ΔdB+2ΔqAfAB(02)·ΔQB+2ΔdA·fAB(12)·ΔQB]

where Δ*q*_*A*_, Δ**d**_*A*_, and Δ**Q**_*A*_ represent the monopole, dipole, and (traceless) quadrupole moments of charge fluctuation on atom *A*, respectively, and

7 $$f_{AB}^{\left(mn\right)}=\frac{1}{m!n!}{\left[\frac{\partial^{m}\partial^{n}}{\partial r^{m}\partial r^{\prime n}}\gamma \left(r,r^{\prime }\right)\right]}_{r=R_{A},r^{\prime }=R_{B}}$$

An on-site approximation is introduced in order to simplify the computation of integrals related to these moments, requiring only the overlap matrix and precalculated atom type specific atomic on-site multipole integrals. The mDFTB3 model extends mDFTB2 with the third-order monopole-based contribution (third term in eq 3) to enhance the description of charged systems.

Benchmark calculations^19^ revealed that the calculated atomic multipole integrals tend to overestimate the total dipole and underestimate the total quadrupole moments. This could be compensated using empirical scaling parameters for the atomic dipole and quadrupole integrals, as shown in ref 19. Since mDFTB2 and mDFTB3 have been mainly tested for noncovalent interactions so far, no generic repulsive potentials have been created yet. The test calculations included neutral hydrogen bonds, repulsive noncovalent interactions, and ionic hydrogen bonds, such as the HB375 and IHB100 data sets^20^ as shown in Figure 1. In addition, the mDFTB2 and mDFTB3 models were tested for treating water clusters (including hydronium and hydroxide), a large water droplet, and a set of proton transfer reactions. For the latter, specific C–H, N–H, and O–H repulsive potentials were developed using a simple training set that included CH_4_, NH_3_, and H_2_O.

**Figure 1:**
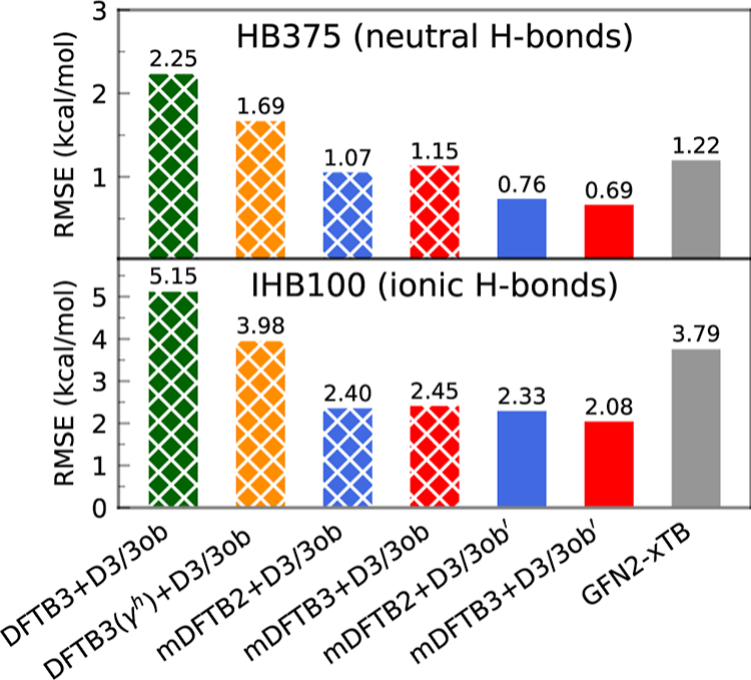
Representative benchmarking results for noncovalent binding energies using the HB375 and IHB100 test sets, comparing mDFTB2, mDFTB3, DFTB3, DFTB3­(γ^*h*^), and GFN2-xTB models. (Reproduced from ref 19. Copyright 2023 American Chemical Society).

In general, even using existing electronic parameters from the monopole-based DFTB3 model, including the multipolar contributions considerably improved the description of noncovalent interactions and proton transfers for both neutral and ionic systems. As an example, the DFTB3 model (with D3-dispersion correction and hydrogen-bonding correction) delivers a root-mean-square error (RMSE) of 1.69 kcal/mol for HB375 and 3.98 kcal/mol for IHB10. The corresponding values are 1.15 and 2.45 kcal/mol when using the mDFTB3 model, while with empirically scaled atomic multipolar integrals, the RMSE values are further improved to 0.69 and 2.08 kcal/mol.^19^

In order to make the multipolar models generally applicable, systematic optimization of the repulsive potentials is still necessary. Furthermore, the creation of mDFTB-optimized electronic parameters might be necessary in the future, as highlighted by the observed trend of overbinding in anionic hydrogen bonds and under-binding in cationic hydrogen bonds.

## 4 Constrained Ground States

DFT and, by extension DFTB and xTB, obtain the ground state of a system in the presence of an external potential. By modifying this potential, it is possible to obtain different ground states with specific properties, such as a chosen charge density (or population).^21^ More generally, various functionals of the system’s single-particle density matrix (*P*) can be constrained by introducing a generalized free energy for the system in the form of a Lagrangian

8 $$F\left[P,\lambda \right]=E\left[P\right]+\sum_{i}\lambda_{i}\left(C_{i}\left[P\right]-C_{i}^{0}\right)$$

where *C*_*i*_[*P*] represents the *i*th constraint functional of the density matrix with a target value of *C*_*i*_^0^. In this formulation, the free energy, *F*, is maximal in the undetermined multipliers λ_*i*_, but minimal (or stationary for DFTB1) in *P*. This approach has been previously applied to different angular quantum numbers of the 4*f* shell of rare-earth impurities,^23^ and is reintroduced for Mulliken charges in the current code-base. Figure 2 shows an example of combining constraints with xTB. This mirrors the density-functional calculation of ref 22, but uses the sum of the Mulliken charges on each of the two bacteriochlorin complexes as targets (marked on the figure), with the net charge transfer between the left zinc complex and the right hydrogenated arms shown on the *x*-axis of the figure. At each constrained amount of charge transfer, the geometry is relaxed to a force tolerance of 1 × 10^–4^ a.u. for each atom and electronic constraints to a gradient of <1 × 10^–6^ a.u.

**Figure 2:**
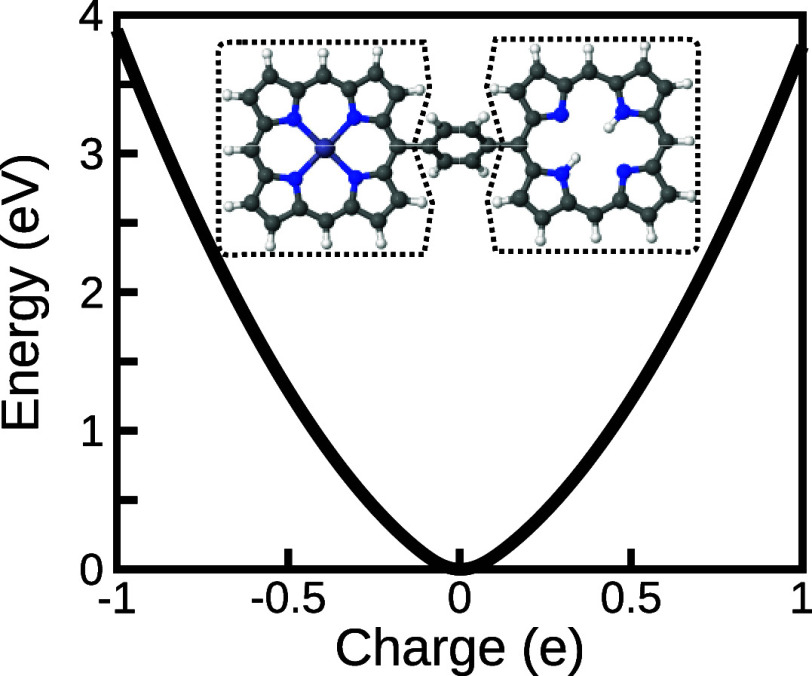
Internal energy of diabatic charge constraints for a GFN2-xTB model of the zinc–bacteriochlorin–bacteriochlorin complex.^22^ The two groups of constrained atoms are surrounded with dotted lines and the net charge of each region is shown on the *x*-axis.

Furthermore, employing an appropriate optimizer, like the nonenergy based optimization method FIRE,^24^ enables the enforcement of constraints even in situations where the Hellmann–Feynman theorem does not hold, such as in transport calculations with open boundary conditions (Section 12). In these cases, even with the nonvariational nature of the system near to the contact regions, the extrema of the functional (eq 8) still correspond to enforcement of the constraint(s).

## 5 Hybrid Functionals

Hybrid functionals, admixing Hartree–Fock (HF)-type exchange with the density functional approximation (DFA), are currently popular choices to address the delocalization problem of Kohn–Sham DFT.^25^ General range-separated long-range corrected hybrid functionals adopt a Coulomb-attenuating method (CAM)^26^ partitioning

9 $$\frac{1}{r}=\underset{DFA}{\underset{︸}{\frac{1-\left(\alpha +\beta \right)+\beta e^{-\omega r}}{r}}}+\underset{HF}{\underset{︸}{\frac{\alpha +\beta \left(1-e^{-\omega r}\right)}{r}}}$$

for the nonlocal exchange operator. This significantly improves various property predictions and spectroscopic observables in particular.^27^ Here, α and β denote the global and long-range fractions of HF-type exchange, mediated by a smooth range-separation function of Yukawa type with a range-separation parameter ω. Niehaus and Della Sala^28^ introduced the LC-DFTB formalism of purely long-range corrected (α = 0, β = 1) hybrid functionals for molecules, as implemented and benchmarked in the DFTB+ package by Lutsker and co-workers.^29^ The latter study found quadratic scaling of the Hamiltonian construction with system size using integral prescreening techniques. Note that the partitioning of eq 9 accommodates conventional semilocal functionals such as PBE^30^ and global hybrids such as PBE0 ^31,32^ as a special case. In the context of the DFTB method they are respectively referred to as PBE-DFTB and PBE0-DFTB in the following.

### 5.1 Two-Component (Noncollinear) Spin

The hybrid functionals are also readily generalized to two-component spinor Hamiltonians, with the implementation so far restricted to molecular systems. As with previous two-component DFTB1–DFTB3,^33^ the density matrix fluctuations, overlap and CAM kernel all take on a spin superblock form

10 $$\Delta \mathrm{P̃}=\left(\begin{array}{ll} \Delta P_{↑↑} & \Delta P_{↑↓}\\ \Delta P_{↓↑} & \Delta P_{↓↓} \end{array}\right),,\mathrm{S̃}=\left(\begin{array}{ll} 1 & 0\\ 0 & 1 \end{array}\right)⊗S,,{\mathrm{\gamma ̃}}^{CAM}=\left(\begin{array}{ll} 1 & 0\\ 0 & 1 \end{array}\right)⊗\gamma^{CAM}$$

where a tensor product is taken between the spin-free terms and the Pauli identity matrix.

### 5.2 Periodic Systems

Reference 34 extended the hybrid DFTB formalism to CAM partitioning and periodic boundary conditions for arbitrary *k*-points. Dielectric-dependent hybrid DFTB has recently been employed to study phonon-induced band gap renormalization in group-IV semiconductors^35^ and benchmarked for simple bulk material band-gaps,^36^ for example the PBE0-DFTB band structure of GaN shown in Figure 3.

**Figure 3:**
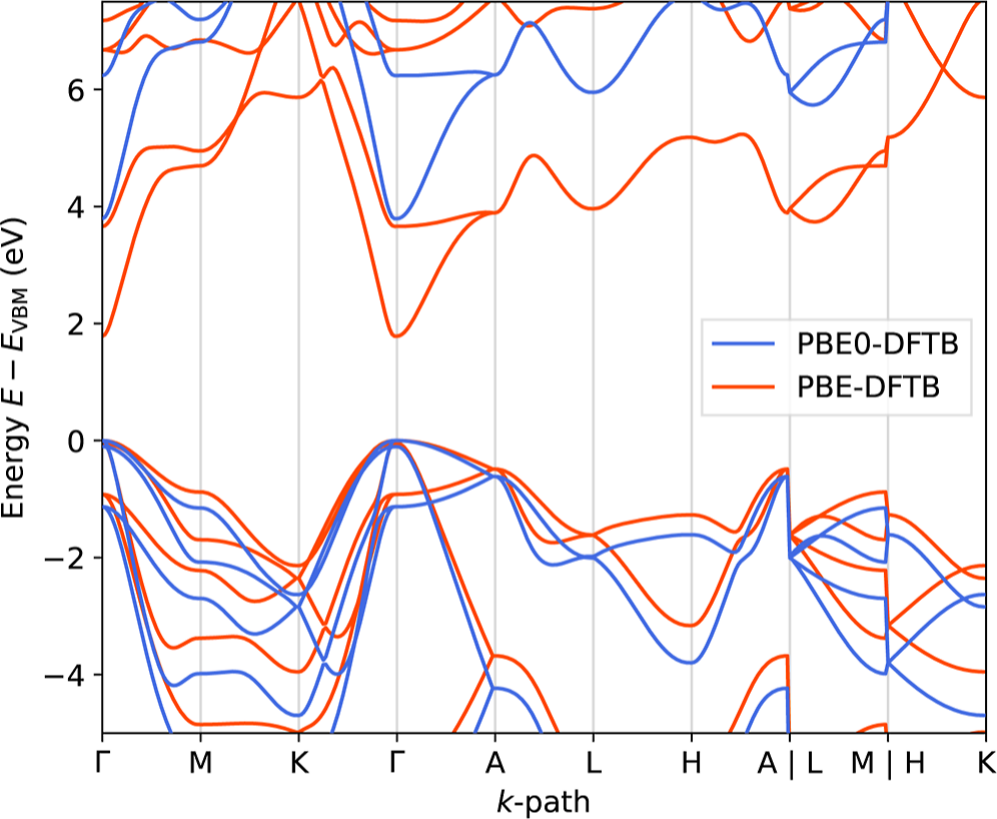
Band structure of the primitive GaN (wurtzite) cell at the experimental lattice constants of *a* = 3.19 Å and *c* = 5.19 Å,^37^ computed on the PBE- and PBE0-DFTB levels of theory. The hybrid PBE0 ^38^ admixes a global fraction of 25% HF-type exchange, which opens the gap from 1.8 eV (PBE-DFTB) to 3.8 eV (PBE0-DFTB), significantly improving the agreement with zero-point renormalization corrected measurements of about 3.6 eV.^37^

CAM-DFTB changes the zeroth-order Hamiltonian and overlap during parametrization but also requires efficient evaluation of HF-type exchange expressions at runtime^34^

11 ΔHμνx,CAM(k)=−18∑λκ∑ghlΔPλκ(g+h−l)Sλμ(h)Sκν(l)×[γμνCAM(g)+γμκCAM(g−l)+γλνCAM(g+h)+γλκCAM(g+h−l)]e−ikg

The density matrix fluctuation Δ*P*_μν_ = *P*_μν_ – *P*_μν_^0^ is with respect to a reference density **P** ^0^ of noninteracting atoms. We adopt the convention that the real-space lattice shifts **g**, **h**, and **l** act on the first orbital, while the second remains in the central cell. γ_μν_^CAM^ is a CAM kernel modified γ-function.^34^ The corresponding energy contribution reads

12 $$E_{x}^{CAM}=\frac{1}{2}\sum_{k}w_{k}\sum_{\mu \nu }\Delta H_{\mu \nu }^{x,CAM}\left(k\right)\Delta P_{\nu \mu }\left(k\right)$$

with *k*-point weights, *w*_**k**_, normalized as ∑_**k**_ *w*_**k**_ = 1. DFTB+ features both a sparse neighbor-list based evaluation of eq 11 in real-space^34,36^ and a dense matrix–matrix operations based algorithm in reciprocal-space. The integrable Coulomb singularity of periodic exchange is treated by either a truncated Coulomb interaction^39^ or Wigner–Seitz truncated density matrix fluctuations (inspired by the minimum image convention of Tymczak et al.^40^). For lower-dimensional systems, the real-space algorithm exhibits near linear scaling,^36^ while the *k*-space algorithm always scales cubically. Colinear spin-polarization is also supported. Analytic energy gradients are available and ongoing work on the stress tensor expression will be reported elsewhere.

## 6 Time-Dependent DFTB

The linear response formulation of time-dependent DFT (TD-DFT) obtains electronic excited states and response properties from the RPA equations^41–43^

13 $$\left(\begin{array}{ll} A & B\\ B & A \end{array}\right)\left(\begin{array}{l} X\\ Y \end{array}\right)=\Omega \left(\begin{array}{ll} l & 0\\ 0 & -l \end{array}\right)\left(\begin{array}{l} X\\ Y \end{array}\right)$$

where the eigenvectors **X**, **Y** determine the transition density and oscillator strength of the excited state of energy Ω. We denote general molecular orbitals (MO) with the indices {*p*, *q*, ...}, occupied orbitals with indices {*i*, *j*, ...}, and virtual (unoccupied) orbitals with indices {*a*, *b*, ...}. The matrices **A** and **B** are^41^

14 Aiaσ,jbσ′=δijδabδσσ′ωjbσ′njσ′−nbσ′+Kiaσ,jbσ′Biaσ,jbσ′=Kiaσ,bjσ′

where ω_*jb*σ′_ = ϵ_*b*σ′_ – ϵ_*j*σ′_ with the occupation numbers *n*_*i*σ_ > *n*_*a*σ_ and *n*_*j*σ′_ > *n*_*b*σ′_.

Within the DFTB formalism, the coupling matrix **K** can be simplified using transition Mulliken charges^44^

15 $$\Delta q_{A}^{ia}=\frac{1}{2}\left(\sum_{\mu \in A}\sum_{\nu }c_{i\mu }^{*}S_{\mu \nu }c_{a\nu }+c_{i\nu }^{*}S_{\nu \mu }c_{a\mu }\right)$$

leading to significant computational savings

16 $$K_{ia\sigma ,jb\sigma^{\prime }}=\sum_{A}\sum_{B}q_{A}^{ia\sigma }\gamma_{AB}^{\sigma \sigma^{\prime }}q_{B}^{jb\sigma^{\prime }}-x_{c}\delta_{\sigma \sigma^{\prime }}q_{A}^{ij\sigma }\gamma_{AB}^{CAM}q_{B}^{ab\sigma^{\prime }}$$

where $\gamma_{AB}^{\sigma \sigma^{\prime }}$ includes the coulomic (γ) and on-site spin resolved interactions (spins σ and σ′) between atoms *A* and *B*.^44^ The prefactor *x*_c_ allows discrimination between TD-DFTB based (semi)­local exchange–correlation functionals^45^ (*x*_c_ = 0) and TD-LC-DFTB (*x*_c_ = 1 and including a fraction of nonlocal Hartree–Fock exchange).^44^ In the former case, the RPA equations are hermitian eigenvalue problems, while hybrid functionals are nonhermitian.^46^ For further information on the accuracy, benefits and drawbacks of TD-DFTB and TD-LC-DFTB compared to TD-DFT and other excited state quantum chemical methods, we refer the reader to ref 47 and several benchmarks from the literature.^44,48–51^

The RPA matrix sizes scale with the product of the number of occupied and virtual orbitals and can be very memory demanding. To address this, we recently parallelized the diagonalization, distributing the RPA vectors (**X** ± **Y**) across the available CPUs. Iterative solution uses either ARPACK-NG diagonalization^52,53^ or an in-house implementation of the Stratmann solver.^46^ The speedup with respect to serial execution for a system with 300 atoms (and response matrix dimension 140,625) is shown in Figure 4. ARPACK-NG provides excellent scaling with a code fraction of roughly 97% running in parallel, while the internal Stratmann diagonalizer is currently much less efficient.

**Figure 4:**
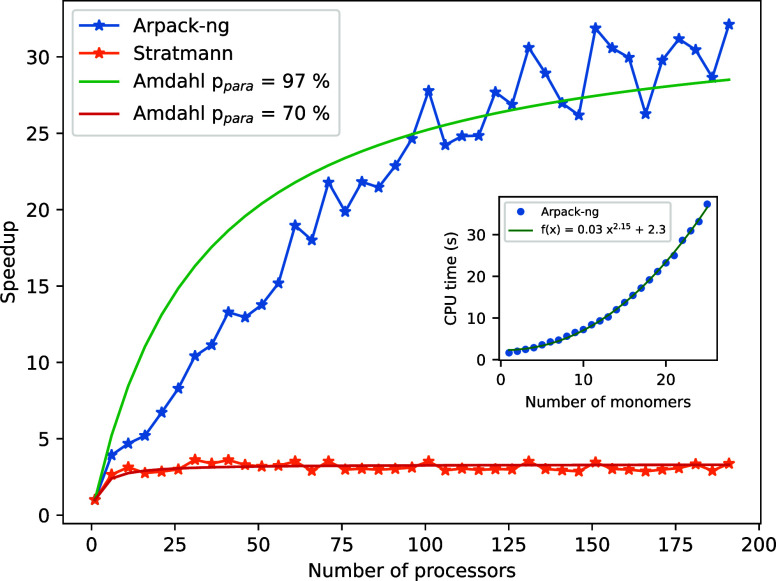
Strong computational speedup with respect to serial execution as a function of the number of processors for a system of 25 stacked benzene molecules (300 atoms) using ARPACK-NG and Stratmann algorithms, compared with Amdahl’s law.^54^ The distance between monomers is 3.0 Å and atomic positions have been randomized by 0.1 Å with respect to the DFTB optimized monomer geometry. Computations of the lowest 100 excited states were performed using TD-DFTB with the mio-1-1 parameters^3^ on AMD Genoa EPYC 9654 2.4 GHz processors. Timings for the lowest 100 excitations states as a function of the number of benzene monomers, using a fixed number of 192 cores and ARPACK-NG, is shown inset.

Additionally, the Tamm–Dancoff approximation (TDA)^55^ has also been recently implemented within the framework of linear response TD-DFTB. This approximation sets the matrices **B** and **Y** of eq 13 to zero. Our implementation supports both TD-DFTB and TD-LC-DFTB. Although the full TD-DFT formulation yields more accurate transition dipole moments for ground to excited state transitions,^56^ the TDA typically maintains good performance for excitation energies and mitigates the effects of triplet instabilities (see, for example, ref 57).

### 6.1 Nonadiabatic Coupling Vector and Conical Intersections

Nonadiabatic molecular dynamics can be used to simulate the coupled dynamics of electrons and nuclei beyond the Born–Oppenheimer approximation. Without time-dependent external fields, nuclear motion drives transitions from one electronic state to another. The probability of such jumps depends on the nonadiabatic coupling vector (NACV)

17 $$d_{nm}^{\xi }=\left\langle \Psi_{n}\left|\frac{d}{d\xi }\right|\Psi_{m}\right\rangle$$

for the electronic states *n* and *m* and a nuclear coordinate, ξ.^58^ In TD-DFT, this quantity can be computed in linear response for ground-to-excited state couplings^59^ and higher order response theory for excited-to-excited state couplings.^60^ The corresponding equations at the DFTB level have recently been derived^61,62^ and their implementation is now available in DFTB+. They include orbital relaxation with a numerical cost very similar to the evaluation of an excited-state gradient. Figure 5 shows the NACV of furan close to a degeneracy between two excited states of the molecule. Such a conical intersection (CI) between two potential energy surfaces provides an efficient channel for internal conversion. We implemented the CI finder by Bearpark et al.^63^ in DFTB+. Integrating the NACV along a path encircling the CI leads to a Berry phase very close to its exact value of π. This result is not obtained for the CI between ground and excited state, due to the well-known difficulties of TD-DFT and TD-DFTB to provide the correct topology of the intersection.^64^ It may, however, be obtained with the REKS-method described in Section 9.

**Figure 5:**
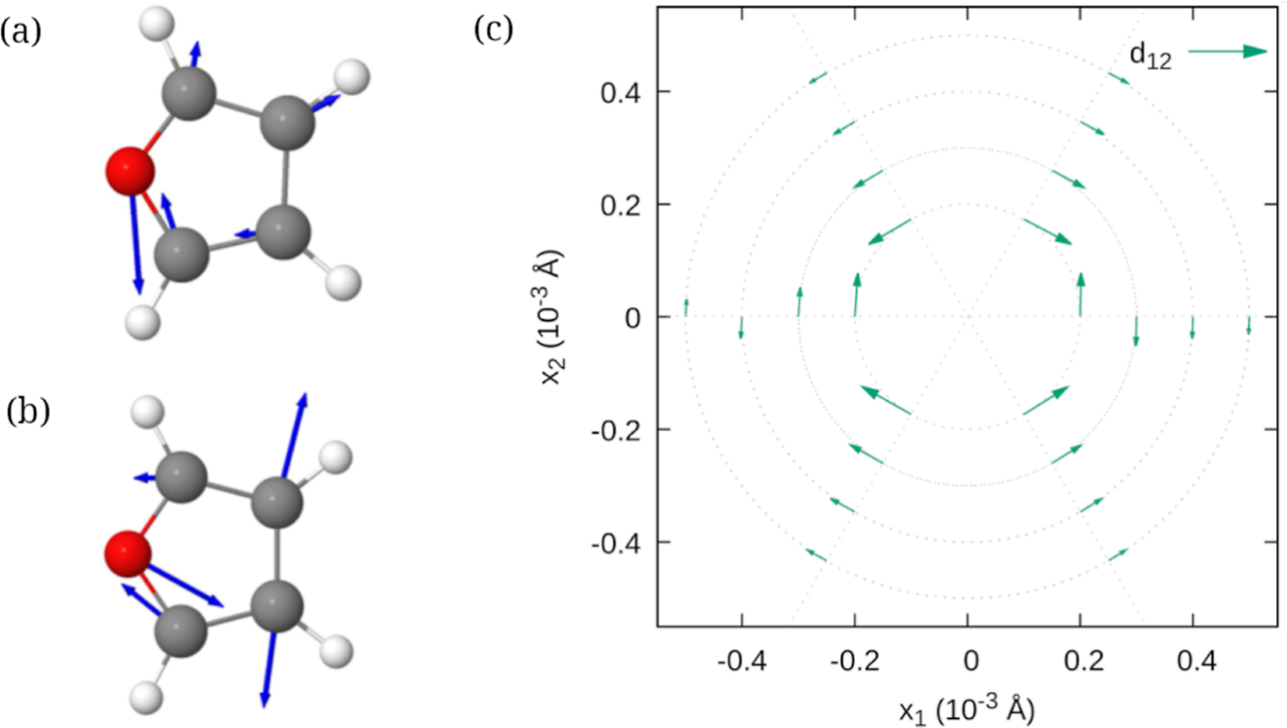
Derivative coupling vectors **d**_12_ for furan *S*_2_/*S*_1_ near the CI as calculated with TD-DFTB. Subfigures (a) and (b) show the geometry and derivative coupling vectors (blue arrows) at different points at a distance of *r* = 2 × 10^–4^ Å from the CI for (a) ϕ = 0° and (b) ϕ = 240°. The coupling vectors are 3*N* dimensional, with *N* the number of atoms, and depicted here as collection of three-dimensional vectors attached to the respective atom. Subfigure (c) shows the components of the coupling vectors in the branching plane of the CI for selected angles along circular paths with radii 2 × 10^–4^ Å to 5 × 10^–4^ Å. (Reproduced with permission from ref 62. Copyright 2023 AIP Publishing).

NACV are also available in the Tamm–Dancoff approximation. The TDA can partially compensate for local density approximation errors, particularly near conical intersections,^65^ making this approach valuable for the simulation of nonadiabatic molecular simulations. We have used this approximation for the simulation of the photodynamics of molecular motors,^66^ with further implementation details provided in a future publication.

## 7 Nonadiabatic Real-Time Electron Dynamics

The excited-state properties of finite systems can be accessed in DFTB+ using the real-time TD-DFTB method, which couples the system to an electromagnetic field under the dipole approximation with a length-gauge Hamiltonian (see ref 67 for a detailed discussion of this formalism). This approach enables the calculation of absorption spectra as well as the study of photoinduced processes by simulating the propagation of electronic states under external fields. DFTB+ supports both purely electron dynamics with fixed nuclei and semiclassical Ehrenfest electron-nuclear dynamics. For the latter, the equation of motion (EOM) for the density matrix *P* is expressed as

18 $$Ṗ=-i\left(S^{-1}HP-PHS^{-1}\right)-\left(S^{-1}DP+PD^{†}S^{-1}\right)$$

where *D* is the nonadiabatic coupling matrix, defined as $D_{\mu \nu }={Ṙ}_{B}\cdot \nabla_{B}S_{\mu \nu }$ . The first term represents the electronic EOM, expressed as a generalized commutator in a nonorthogonal basis.^68^ The second term accounts for nonadiabatic interactions, facilitating energy exchange between electrons and nuclei at the Ehrenfest level. This method has been successfully applied to finite systems to investigate optical properties and photoinduced charge transfer, as demonstrated in studies on chromophore-labeled gold nanoclusters,^69,70^ among other systems.

### 7.1 Real-Time Electron Dynamics in Velocity Gauge

For periodic systems, the length gauge can only be applied when the electric field polarization has components exclusively in a nonperiodic direction,^67^ allowing the definition of the position operator. As examples of this strategy, the study of Fano-induced transparency in sensitized graphene nanoribbons^71^ or electron photoinjection in ZnO nanowires^72^ can be mentioned. Fully periodic systems and perturbations aligned with periodic directions remain unfeasible within the length gauge framework, though. To overcome these limitations, we utilized the gauge freedom inherent to classical electromagnetic fields and adopt the velocity gauge framework. We employed the Peierls substitution method,^73,74^ which has been successfully applied in various contexts already, such as non-self-consistent Ehrenfest dynamics^75^ and self-consistent time-dependent tight-binding calculations.^76^ The method involves modifying the elements of the unperturbed Hamiltonian matrix, *H*_μν_^0^, a follows

19 $$H_{\mu \nu }\left(t\right)=H_{\mu \nu }^{0}\;\exp\left(-\frac{i}{2\pi }A\left(t\right)\cdot \left(R_{\mu }-R_{\nu }\right)\right)$$

where **A**(*t*) is the vector potential and **R**_κ_ denotes the position of the atom containing the orbitals κ. We apply the long-wavelength approximation by neglecting the spatial variation of the electromagnetic field, resulting in a vector potential that depends only on time.

As an initial step toward implementing real-time TD-DFTB fully coupled with the total (external and induced) vector potential, we introduced the calculation of the current density in the code. Within the DFTB framework, the spatially dependent current density is described in terms of orbital or interatomic currents. For finite systems, the current density expression can be derived from the continuity equation using the tight-binding formalism.^77^ In the context of nonorthogonal tight-binding, bond currents can be defined through projector operators and by considering the rate of change of the number of electrons.^78^ Although this definition is not unique in a nonorthogonal basis, it satisfies the continuity equation, integrates to the correct physical quantity and provides valuable insights into the emerging electrodynamics. The expression for the current between orbitals μ and ν implemented in the code follows ref 79

20 $$J_{\mu \nu }=-\left\{H_{\mu \nu }Im\left(P_{\mu \nu }\right)-S_{\mu \nu }Im\left(E_{\mu \nu }\right)\right\}$$

where *E* is the energy weighted density matrix, satisfying the relation *HP* = *SE*. To resolve the vector components along the Cartesian directions, the current density is projected onto a unit vector along each bond direction: $J_{AB}=\sum_{\mu \in A}\sum_{\nu \in B}J_{\mu \nu }\frac{R_{AB}}{\left|R_{AB}\right|}$ . Figure 6 illustrates an example of the application of these time-dependent bond currents. When a Mg-porphyrin molecule is irradiated with circularly polarized laser light, a rotating current can be induced, and it is even possible to prepare current-carrying stationary states.^80^ The charge density, as evidenced by the electrostatic potential $V_{el}\left(r\right)=\sum_{A}\frac{\Delta q_{A}}{\left|r-R_{A}\right|}$ , rotates in the same direction as the incident pulse. For visualization purposes, the bond currents are transferred onto the same spatial grid used for plotting the electrostatic potential by placing Gaussian functions at the midpoints point between each pair of atoms, with a width of σ = 0.5 Å. The current distribution clearly follows the spatial profile of the charge density difference.

**Figure 6:**
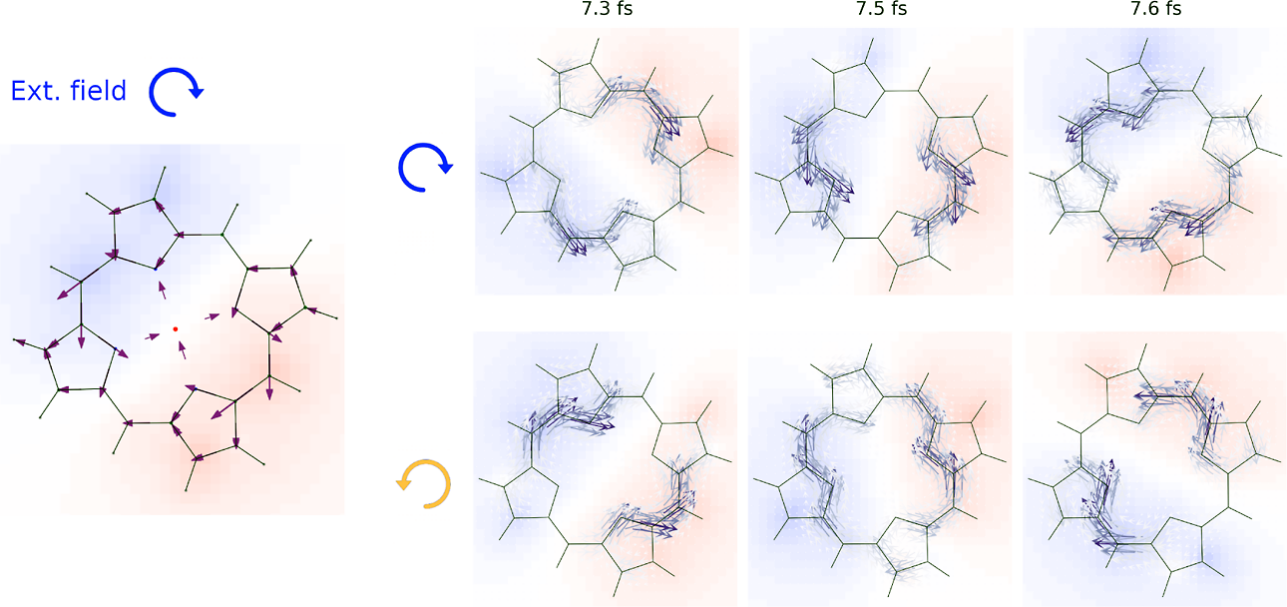
A Mg-porphyrin molecule oriented in the *xz* plane is irradiated with circularly polarized light. The bond currents calculated from eq 20 are shown in the left panel. The circularly polarized pulse induces a rotating charge distribution with an associated current density. The current can be transferred to a grid for better visualization (right panels) evidencing the opposite direction of rotation with left and right polarization. Furthermore, the current has its maximum values in the spatial region with the maximum charge difference with respect to the ground state. The charge density is depicted indirectly by plotting the electrostatic potential it generates (red being positive and blue negative potential respectively).

As an example for the periodic velocity-gauge implementation, we present the study of graphene’s population dynamics in reciprocal space. This study mimics the irradiation conditions employed in refs 81 and 82 , where the anisotropic optical absorption of graphene was addressed within the Fermi golden rule framework. A snapshot of the dynamics at ∼96 fs along the *K*–*K*′ path is shown in Figure 7a, where the changes in the population compared to the ground state can be observed. A laser of energy *h*ν = 3 eV, as in ref 81, was used. Our real-time approach captures the dynamics of electronic density promotion beyond the Fermi level. As time evolves, the population/depopulation features become localized around the 3 eV threshold. Note that trigonal warping is expected at high excitation energies. The observed energy and *k* dispersion emerge intrinsically from the dynamics and can be understood in terms of the density of states and the time-dependent oscillatory component of the transition probability in the Fermi golden rule (see ref 72 for details). Figure 7b,c present the 2D *k*-dependent population increase in graphene’s conduction band for laser polarization aligned along the *x* and *y* axes, respectively. These results are in close agreement with those reported in refs 81 and 82 . Population nodes aligned with the *x* and *y* directions appear as a function of laser polarization (see dashed lines), closely resembling the nodes predicted from the *k*-dependent absorption cross-section.

**Figure 7:**
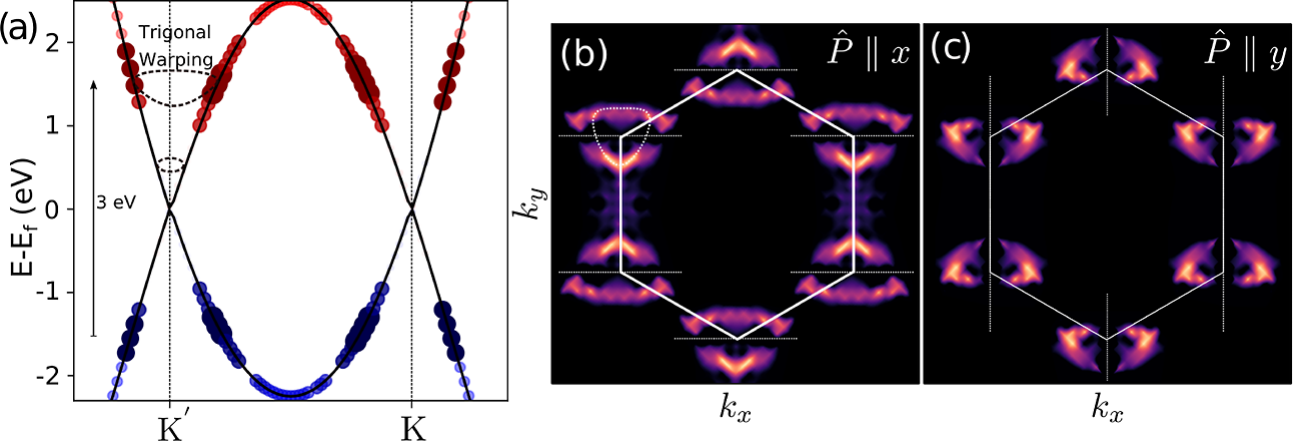
*k*- and time-dependent absorption of graphene as an example of the velocity gauge implementation. (a) Snapshot of the time-dependent populations at ∼96 fs in the *K*–*K*′ path. Red/blue dots depict the population/depopulation dynamics, respectively. The dot color, size, and opacity encode the population change. The arrow indicates the excitation energy, while the trigonal warping is highlighted with dashed lines. (b,c) Population pattern in *k*-space as a function of laser polarization.

### 7.2 Parallel Implementation of the Electron Dynamics

One of the most widely used applications of the real-time electron dynamics implemented in DFTB+ is the study of light-induced dynamics in nanoscale systems, particularly electron–ion dynamics in irradiated plasmonic nanoparticles. Numerous studies have explored various aspects of these phenomena, including linear spectroscopy,^83–85^ and high harmonic generation,^86^ as well as plasmon-induced hot carrier generation and its photocatalytic potential.^87,88^ However, advancing the field further requires studying of atomistic systems consisting of several thousand atoms. This would open the door to exciting avenues of research in plasmonics, polaritonics, catalysis, spectroscopy and light shaping, among other areas.

The recently implemented parallelization of the linear algebra operations of the electron dynamics module (using the Message Passing Interface, MPI,^89^ framework) enables the study of even larger finite systems at the nanoscopic scale and addresses the RAM limitations of single-node calculations (this implementation supports the hybrid functionals of Section 5). Figure 8 shows a sequence of absorption spectra for icosahedral silver nanoparticles with sizes ranging from 147 to 1415 atoms. This series, originally presented in ref 83, represents, to the best of our knowledge, the largest published electron dynamics study conducted using this method (noting that modern computational resources can now handle even larger systems). To illustrate the capabilities of the MPI parallelization, the spectra of particles containing 5083 and 8217 atoms are also shown in the figure. The inset shows the evolution of plasmon energy as a function of the inverse cubic root of the number of atoms in the nanoparticle. As anticipated, the plasmon energy exhibits a linear relationship with the particle’s surface-to-volume ratio.

**Figure 8:**
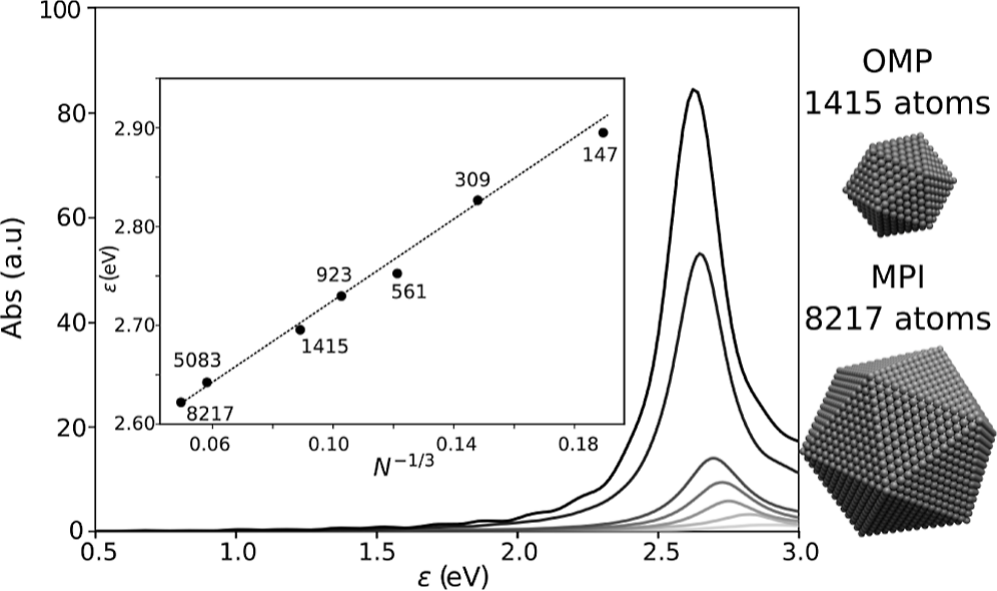
Absorption spectra of silver nanoparticles consisting of 147, 309, 561, 923, 1415, 5083, and 8217 atoms, respectively. The inset shows the linear trend followed by the energy of the plasmonic resonance as a function of the particle surface to volume ratio. Results for the two largest particles were obtained by utilizing the MPI-parallelization, while OpenMP (OMP) parallelization was used for the smaller particles.

## 8 Time-Independent Excited States

The DFTB+ package also includes an implementation^2^ of ΔSCC-DFTB (often shortened to ΔDFTB), a time-independent approach to the energy and properties of the lowest-lying singlet excited state.^90^ The singlet excited-state energies are calculated from independent SCC-DFTB calculations of the system in two distinct electronic configurations: the lowest-energy triplet determinant with energy *E*_t_, and a determinant in which one electron is promoted from the highest occupied molecular orbital (HOMO) to the lowest unoccupied molecular orbital (LUMO). The latter determinant, with energy *E*_m_, is not a spin eigenfunction, but the singlet excited-state energy can be approximated by calculating *E*_m_ and *E*_t_ through separate SCC-DFTB calculations with different sets of unrestricted orbitals and then applying the Ziegler sum rule,^91,92^ *E*_s_ = 2*E*_m_ – *E*_t_. ΔDFTB provides convenient and efficient access to excited-state geometries and frequencies because the determinants are effectively ground state determinants under occupation constraints.

Transition properties such as the transition dipole moment (TDM) between ground and excited states cannot be directly extracted from a standard ΔDFTB calculation. Unlike TD-DFTB, where excited states are expressed in terms of transition vectors relative to a reference ground state, ground and excited states in ΔDFTB are optimized separately. DFTB+ can evaluate the TDM between the DFTB ground state and ΔDFTB excited state for nonperiodic systems.^93^ Similarly to the case for TD-DFTB, the TDM between ground and excited states is expressed in terms of atomic transition charges Δ*q*_*A*_^*ia*^ (eq 15). In the case of ΔDFTB, however, there is an ambiguity in the definition of the MO coefficients, **C**, because the SCC-DFTB ground state and the ΔSCC-DFTB excited state are separately variationally optimized, so there is no guarantee that ground- and excited-state optimized MOs are orthogonal (i.e., their determinants are not necessarily from the same set of MOs). This ambiguity is addressed in DFTB+ through a corresponding orbital transformation (COT)^94,95^ that rotates the optimized ground and excited state orbitals into an optimally mutual orthogonal basis.^93^ The resulting rotated orbitals are used in place of the MO coefficients **C** in eq 15 to evaluate the TDM.

Benchmarking of ΔDFTB TDMs against TD-DFT for small organic molecules demonstrates^93^ that the accuracy of the TDM orientation is very sensitive to the suitability of the HOMO → LUMO approximation implicit in the construction of the mixed-spin ΔDFTB determinant: orientations are close in cases where the approximation is reasonable and deviate significantly in cases where the approximation is unsuitable, e.g., due to dominant HOMO – 1 → LUMO character. Further benchmarking on acridinium photocatalysts^93^ demonstrates the potential utility of ΔDFTB for high-throughput virtual screening of changes in the TDM orientation of molecular photocatalysts as functional groups are added or removed.

## 9 REKS Improvements for Excited State Calculations

Instead of using single determinants (or only two as in ΔDFTB), the spin-restricted ensemble-referenced Kohn–Sham (REKS) and its state-averaged (SA-REKS) and state-interaction (SI-SA-REKS, or SSR) variants^96–101^ based on ensemble density functional theory, are also supported in DFTB+. The original hybrid-DFTB based SSR implementation (LC-DFTB/SSR)^102^ can describe the ground and lowest excited state with a long-range corrected hybrid functional. However, it shows different stability depending on excitation characters for open-shell singlet microstates. This can be improved with simple scaling of the spin-polarization parameters,^102^ but to improve energy calculations without artificial treatment, onsite correction to the two-electron integrals can be helpful for such excitations. The original implementation of onsite corrections has been applied to a full-range Hartree exchange–correlation kernel, *f*_*Hxc*_, namely full-range onsite correction (frOC).^103^ In particular, the onsite correction to a long-range Hartree–Fock exchange kernel, *f*_*x*_, yields the additional energy term (i.e., long-range onsite correction, or lrOC)

21 E2ndlr,ons=−14∑σ∑μνμ≠ν∑κλ(ΔPμλσΔPκνσ+ΔPμνσΔPκλσ)SμλSκν(μν|fx|μν)lr−14∑σ∑μνμ≠ν∑κλ(ΔPμλσΔPμκσ+ΔPμμσΔPκλσ)SνκSνλ(μν|fx|μν)lr

where ${\left(\mu \nu \left|f_{x}\right|\mu \nu \right)}^{lr}$ denotes the integral of the orbitals μ and ν with the long-range Hartree–Fock exchange kernel. These onsite corrections to LC-DFTB/SSR (LC-OC-DFTB/SSR) improve excitation energies and correctly describe CI geometries with standard parametrizations.^104^ Figure 9a,b show the optimized structures at the CI between ground and lowest excited states with reasonable accuracy for π/π* or *n*/π* transition of Feringa’s molecular motor.^105–107^ The strong pyramidalization is observed only if the long-range onsite correction terms, eq 21, are included in the SSR­(2,2) formalism.^104^

**Figure 9:**
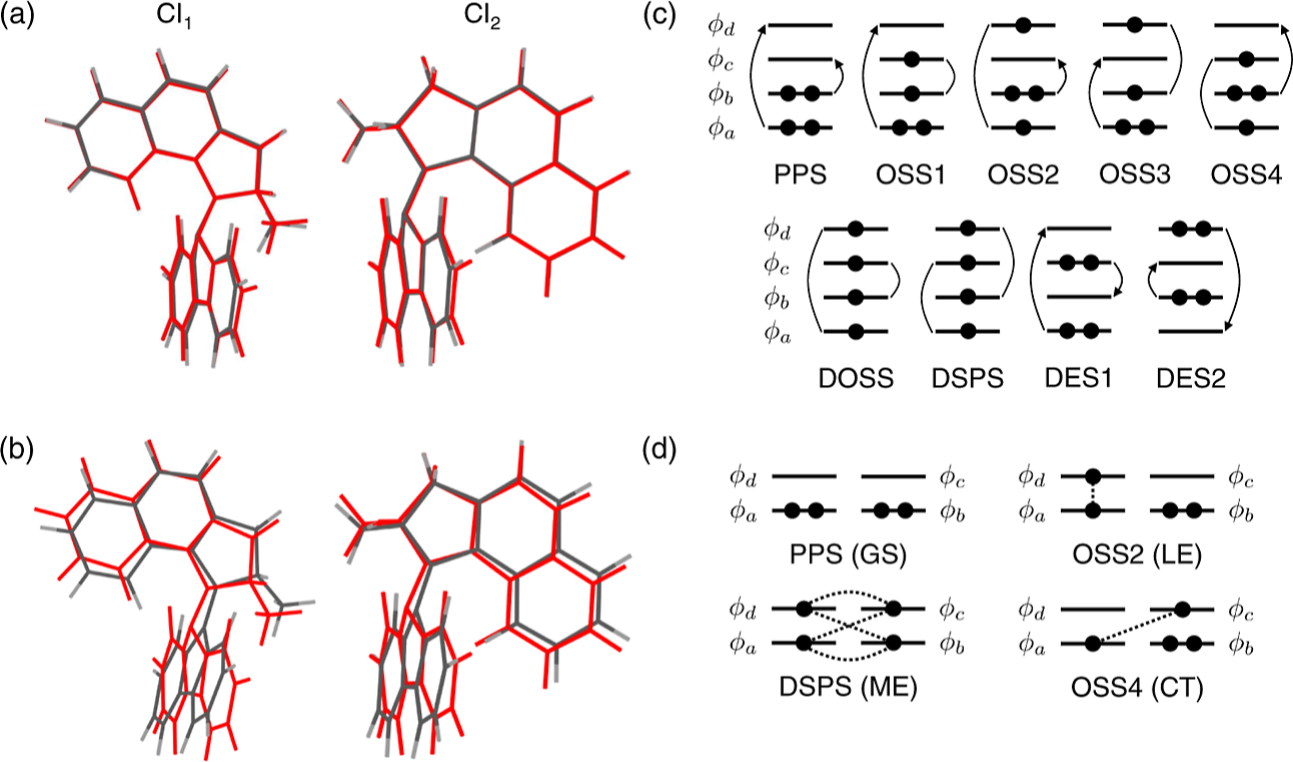
Molecular motor structures at the conical intersection. Red represents SSR/ωPBEh geometries, while (a) LC-OC-DFTB/SSR or (b) LC-frOC-DFTB/SSR structures are in black. The strong pyramidalization near the center C=C bond does not appear without lrOC terms. (c) Schematic representation of the configurations with four active orbitals. Round arrows indicate double excitations, while arrow-less round brackets show open-shell singlet coupling between unpaired electrons. (d) For dimeric systems, the electronic configurations correspond to the ground (GS), locally excited (LE), charge transfer (CT), and multiexcitonic (ME) states. Dashed lines show the open-shell singlet couplings between unpaired electrons.

Recently the DFTB/SSR method with an extended active space of four electrons and four orbitals, DFTB/SSR­(4,4), has been developed to study excitation energy transfer of multichromophore systems.^108^ A total of nine electronic configurations can be obtained from these four active orbitals, corresponding to one perfectly spin-paired singlet (PPS) state, four open-shell singlet (OSS) states, one double open-shell singlet (DOSS) state, one doubly spin-polarized state (DSPS), and two doubly excited states (DESs) (Figure 9c).^108,109^ The contribution of DOSS and DSPS to the ground state becomes significant in situations with dissociating double bonds or for nearly degenerate active orbitals. Excitonic couplings between locally excited states in anthracene and tetracene dimers have been studied with reasonable accuracy from the OSS1–OSS2 coupling in LC-OC-DFTB/SSR­(4,4) method (Figure 9d).^108^

## 10 Implicit Solvents

Solvation effects can be included implicitly by a dielectric medium interacting with atomic charges, either in a surrounding cavity (polarizable continuum model^110^) or an implied cavity (generalized Born (GB)^111–113^ or analytical linearized Poisson–Boltzmann (ALPB)^114,115^ ). For the dielectric contribution δ*G*_diel_ we arrive at the following energy equation

22 $$\delta G_{diel}=f\left(\varepsilon \right)\sum_{A}\sum_{l=0}^{L_{\max}}\sum_{m=-l}^{+l}{\left[\Psi_{A}\right]}_{l}^{m}{\left[X_{A}\right]}_{l}^{m}$$

where *f*(ε) is a function of the dielectric constant ε, *L*_max_ is the maximum angular momentum for the spherical harmonic basis with angular momentum ( l , *m*), Ψ is the charge distribution mapped to the basis, and *X* is the response of the dielectric medium in the given basis. The function *f*(ε) is given as

23 $$f\left(\varepsilon \right)=-\frac{1}{2}\frac{\varepsilon -1}{\varepsilon +\alpha }$$

where α is a fixed value of 0.571214 for finite dielectric constants, following the work of Sigalov,^114^ or zero for the ideal conductor with ε → ∞.

In case of a polarizable continuum we can express our charge distribution in terms of the Mulliken partial charges, *q*_A_, by domain decomposition, ${\left[\Psi_{A}\right]}_{l}^{m}=\sqrt{\pi }q_{A}\delta_{l0}\delta_{m0}$ , where δ_*ab*_ is the Kronecker delta. The solution *X* is obtained by solving the direct polarizable continuum equation: *LX* = *g*, with *L* being the block sparse matrix for the interaction in a spherical harmonic basis and *g* the spherical harmonics expansion of the molecular potential. The molecular potential is evaluated by the Coulombic interaction of the charge distribution with the grid points.

Similar to the polarizable continuum model we can define an analytical linearization of the Poisson–Boltzmann (PB) equation, known as generalized Born model, where the maximum angular momentum is chosen to be zero. The dielectric energy in this case is expressed as

24 $$\delta G_{diel}=f\left(\varepsilon \right)\sum_{A}\Psi_{A}X_{A}$$

where the charge distribution Ψ_A_ = *q*_A_ and we compute *X* from

25 $$X_{A}=\sum_{B}^{N_{atoms}}q_{B}\left(\frac{1}{f\left(R_{AB},a_{A},a_{B}\right)}+\frac{\alpha }{\varepsilon A_{\det}}\right)$$

where *a*_A/B_ are the atomic Born radii and $A_{\det}$ is a model of the electrostatic size of the solute. Two interaction kernels (*f*) have been implemented: the canonical kernel of Still,^113^ or the alternative P16 kernel.^116^ For computing the Born radii several schemes have been proposed, in DFTB+ the Onufriev–Bashford–Case (OBC) corrected integrator termed GB^OBC^II is used.^117^ The main advantage of the ALPB or GB models over PCM is their negligible computational cost, allowing inclusion of solvation effects for minimal increase in evaluation time.

## 11 QM-MM Coupled-Perturbed Response

One of the active application areas of DFTB+ is the study of chemical reactions in extended molecular systems, such as biomolecular complexes. A natural way to enhance the efficiency of such calculations is to employ a hybrid quantum mechanical/molecular mechanics setup (QM/MM) with electrostatic embedding. In this setup, the electron density in the QM region dynamically responds to the charge distribution in the MM region, leading to nonzero derivatives of atomic charges with respect to the coordinates of the MM atoms. Building on previously developed coupled-perturbed expressions for the QM region,^2,118,119^ the nonvariational QM/MM derivatives can now also be evaluated in DFTB+.

Such derivatives contribute to the atomic forces—and thus have to be considered and calculated additionally—whenever biasing potentials are applied to collective variables composed of partial atomic charges in extended sampling QM/MM MD simulations, such as umbrella sampling or metadynamics. This approach is particularly powerful for investigating biomolecular reactions, as it enables the generation of the potentials of mean force for the reaction(s) of interest, from which the underlying microscopic mechanisms may be inferred. We applied this new method to study the proton-coupled electron transfer in a model of the reaction center of proteins of the photolyase and cryptochrome family.^120,121^

## 12 Investigating Phonon-transport

Nanophononics is a relatively young field, driven by the quest for new materials to improve heat flux control at the nanoscale for thermal management in nanoelectronic devices, and for novel mechanisms to improve thermoelectric energy harvesting and recycling.^122^ A key concept in this field is the quanta of phonon heat conductance,^123^ κ_0_ = π^2^ *k*_B_^2^ *T*/3*h*, serving as the thermal counterpart of the well-known quantization of the electronic conductance, *G*_0_ = *e* ^2^/*h*, per spin channel. However, measuring phonon conductance is more challenging than electronic conductance due to the bosonic nature of phonons and the difficulty in imposing well-defined temperature gradients across nanojunctions. Despite these challenges, 2D materials such as graphene, which exhibit long phonon mean free paths at room temperature (≈100–1000 nm), provide promising platforms for investigating thermal transport at the nanoscale.

Phonon transport phenomena can be investigated at the quantum mechanical level within DFTB+ using the recently introduced *phonons* tool. This tool interfaces with the libNEGF library,^124^ which was previously already employed to address electronic quantum transport problems. In this framework, the contour-ordered phonon Green’s function (GF) is defined as *G*_αβ_(*t*, *t*′) = – *i*⟨*T*_c_ *u*_α_(*t*)*u*_β_(*t*′)⟩, where *u*_α_(*t*) represents the atomic displacement from equilibrium at time *t* of atom and direction α, and *T*_c_ is the contour time ordering operator. This GF satisfies the equation of motion (EOM)

26 $$-\frac{\partial^{2}G_{\alpha \beta }\left(t,t^{\prime }\right)}{\partial t^{2}}-\sum_{\sigma }K_{\alpha \nu }G_{\nu \beta }\left(t,t^{\prime }\right)=\delta_{\alpha \beta }\delta \left(t-t^{\prime }\right)$$

where *K* is the harmonic dynamical matrix. While anharmonic effects, such as phonon–phonon and phonon–electron interactions, could be incorporated as additional terms in the EOM (see, for example, ref 125 and references therein), they are currently not considered. The harmonic limit remains valid at low temperatures and provides valuable insights into scattering mechanisms, including grain-boundary defects, interfacial scattering, substrate interactions and scattering at domain boundaries.

For steady-state solutions, the system can be partitioned into a central scattering region and contacting leads (using self-energies, Σ, which can also include interactions). The machinery is formally identical to the electronic GFs

27 $$G^{r}\left(\omega \right)={\left[{\left(\omega +i\delta \right)}^{2}-K-\Sigma^{r}\left(\omega \right)\right]}^{-1}$$

with the dynamical matrix as the Hamiltonian. The retarded Green’s function *G* ^r^(ω) can be used to obtain the coherent transmission between contacts and the thermal conductance by means of a Landauer-like expression

28 $$\kappa_{ph}=\frac{1}{2\pi }\int_{0}^{\infty }\omega Tr\left[\Gamma_{1}\left(\omega \right)G^{r}\left(\omega \right)\Gamma_{2}\left(\omega \right)G^{a}\left(\omega \right)\right]\frac{\partial n_{B}\left(\omega \right)}{\partial T}d\omega$$

where Γ_*j*_ = *i*[Σ_*j*_^r^ – Σ_*j*_^a^] is the contact line width, *n*_B_(ω, *T*) the Bose–Einstein distribution, and the expression in the trace (Tr[...]) defines the ballistic phonon transmission. The phonon tool available in the DFTB+ package can read various types of Hessian matrices, not limited to DFTB calculations, e.g., LAMMPS (Large-scale Atomic/Molecular Massively Parallel Simulator).^126^ The electronic transport features of DFTB+ have been discussed previously.^2,124^ From the *n* moments of the electronic transmission, *L*_*n*_, the electronic conductance, *G* = *e* ^2^ *L*_0_, the Seebeck coefficient

29 $$S=\frac{1}{qT}\frac{L_{1}}{L_{0}}$$

and, via Onsager linear-response relationships, the electronic contribution to the thermal conductance can be obtained

30 $$\kappa_{el}=\frac{1}{T}\left[L_{2}-\frac{L_{1}^{2}}{L_{0}}\right]$$

From these quantities it is possible to obtain the well-known figure of merits for thermoelectric applications, *ZT* = *GS* ^2^ *T*/(κ_el_ + κ_ph_). Applications of the developed code include studying grain-boundary defects in graphene^127,128^ including functionalization,^129^ thermal transport across molecules bridging graphene flakes^130^ and thermoelectric properties of 2D materials.^131,132^

## 13 Improving Density Matrix Efficiency Utilizing GPUs

In the workflow for DFTB ground-state calculations, the two most time-consuming tasks are the diagonalization of the Hamiltonian matrix and the construction of the density matrix. In previous work, we demonstrated that performing the diagonalization step on 6 NVIDIA Tesla V100 GPUs achieved a speedup of 16× with respect to the CPU-only calculation.^2,133^ Scaling of the diagonalization of the Hamiltonian matrix with number of GPUs shows a dependency with the matrix size; the bigger the matrix, the better the scaling.^133^ As the diagonalization process becomes faster, the density matrix construction begins to significantly impact the overall runtime, which leads to the need to also port it to GPUs.

The density matrix, *P*, is built from the eigenvector matrix, *C*, by calling the Hermitian rank-k matrix multiplication update (*HERK*) on *P*, such that *P* ← α*CC* ^*H*^ + β*P*. In our implementation, the CPU-based BLAS level 3 *HERK* routines are replaced with their single-GPU-based counterparts provided by the MAGMA library.^134^ The transfer of the eigenvectors and density matrix between the host and devices, and the memory allocation are managed by handling the GPU pointers directly.

Figure 10 compares wall-clock times for performing the diagonalization and density matrix construction for a water cluster system with 31,680 basis functions. The results indicate that executing both tasks on a single GPU is faster than performing diagonalization alone on two GPUs. Overall, executing both diagonalization and density matrix construction on GPUs leads to a speedup of 1.4× for one GPU and 1.5× for two GPUs with respect to just performing the diagonalization on the GPUs. The time spent in the different routines shows that when both operations are executed on the GPUs, the relative time for constructing the density matrix lowers significantly from 30–40% to 5–10% when compared with only performing the diagonalization on the GPUs. This is consistent with the relative times observed in the CPU-only calculations.^133^

**Figure 10:**
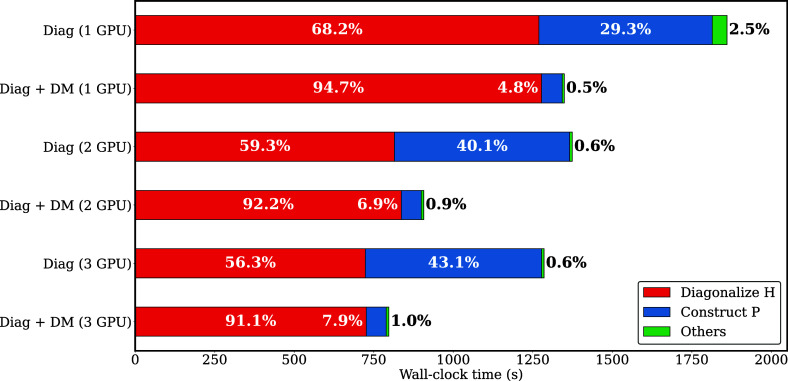
Comparison of the wall-clock time of DFTB single-point energy calculation of a water cluster (H_2_O)_5280_, with a percentage decomposition into the diagonalization of the Hamiltonian matrix *H*, the density matrix *P* construction and other contributions. All calculations were performed on SUMMIT using 21 POWER CPU threads + V100 GPUs. “Diag” denotes the calculations where only the diagonalization was performed on the GPUs, whereas “Diag + DM” represent calculations where both the diagonzalization of *H* and the construction of the density matrix were carried out on the GPUs.

## 14 Generating Parameters for the DFTB Method

There already exist numerous parameter sets for the DFTB method, enabling the calculation of a wide variety of systems. Many of these sets can be found on the dftb.org website^135^ Some sets are rather general (such as 3ob, matsci, ob2, pbc) and cover a wide range of elements and chemical compositions, while others are mostly targeted to describe a narrow selection of systems well. Notably, a recent effort by Cui et al.^136^ resulting in so-called baseline parameters for solids covers nearly the entire periodic table. These parameters can be used out-of-the-box for many systems and can serve as a good starting point for reparameterization if higher accuracy is needed for a smaller selection of elements and systems. In cases where parameters are not available or do not provide the desired accuracy, the DFTB+ software suite offers tools to create new parameter sets.

### 14.1 Electronic Parameters

The open-source parametrization suite SkProgs^137^ provides a complete set of tools for generating the integrals needed to compose the nonscc Hamiltonian *H* ^0^ and the overlap matrix *S* in actual calculations (see Section 2.1.1). With the help of the libxc-library,^138^ it offers a wide choice of possible exchange–correlation functionals, including meta-GGA and range-separated long-range corrected hybrid functionals. The suite comprises three hierarchically ordered components: slateratom, a HF/DFT code for spherically symmetric pseudoatoms; sktwocnt, a dimer integration code for computing the Hamiltonian and overlap matrix elements of high-symmetry orbital configurations; and the sktools, a master utility driving the calculations with the former two as to generate complete sets of Slater–Koster parameters and derived properties such as spin-coupling constants. While the performance critical binaries (slateratom and sktwocnt) are written in modern Fortran, the sktools are implemented in Python. SkProgs is available as an install from source or via the conda package management system through the conda-forge channel.^139^

### 14.2 Repulsive Potentials

To facilitate the computation of total energies and forces, a DFTB parametrization must also include distance-dependent repulsive potentials for each atom type pair (see Section 2.1.1). A common strategy for determining these potentials involves optimizing them to minimize the discrepancy between DFTB-predicted values and reference data for selected physical properties across a specific set of systems. These reference values are typically obtained from ab initio calculations, where energies and forces are extracted, though experimental data can also be used for comparison. The simplest fitting approach involves a single system in which each species–species interaction is represented by a single bond (or multiple symmetrically equivalent bonds). The repulsive potential as a function of distance can be derived by systematically varying the bond length and computing the energy difference between the reference and DFTB values. This process is facilitated by the splinerepfit script within the SkProgs package.

For more complex cases, additional methods and external tools are available. The curvature constrained splines (CCS) method,^140^ for instance, enables simultaneous fitting of multiple repulsive potentials while preventing artificial oscillations by imposing constraints on the second derivative. Another promising approach is the ChIMES force field,^141,142^ which employs Chebyshev polynomials to represent the repulsive potential. It can incorporate higher-order interactions, such as three-body terms, which have been shown to substantially improve accuracy in systems where two-body terms alone are insufficient.^143^ Additionally, machine learning techniques are increasingly being explored as a means to predict the repulsive potential for specific systems.^144–148^

## 15 Communication with External Tools

### 15.1 Integration with LAMMPS

DFTB+ provides a comprehensive application programming interface (API). External programs can include this as a library, trigger calculations within DFTB+, and retrieve results. Recently, the DFTB+ API has been extended to allow for importing the neighbor list from an external source, and thus bypass its computation in DFTB+, resulting in potential performance optimizations. The main motivation for this extension is the development of a plugin for the LAMMPS molecular dynamics software suite.^126^ Here, we provide a custom pair_style command for simulation with DFTB+ to determine the atomic forces, stress tensor components, and total energy of a configuration. This allows for efficient simulation with DFTB+ driven by LAMMPS for a host of ensembles and application areas, including but not limited to *NVT*, *NPT*, dynamic loading, determination of kinetic parameters via the nudged elastic band (NEB) method^149^ and free energy calculations.

### 15.2 Exchanging Dense Matrices with DFTB+

The DFTB+ API is also compatible with the atomic simulation interface (ASI)—an API for deep integration with other codes on the level of electronic structure data, such as Hamiltonian, overlap, and density matrices.^150^ The ASI project provides a lightweight wrapper library that maps a subset of DFTB+ API functions to the ASI API naming convention. This design keeps the DFTB+ API uniform, while maintaining compatibility with other tools that are based on ASI, including the asi4py Python wrapper and the FHI-aims DFT code^151^ that also implement ASI.

In order to avoid making unnecessary copies, the ASI uses callbacks providing direct access to the raw data in memory. Users can register callback functions to pass from, or receive, large matrices from DFTB+ (Hamiltonian, overlap and density matrices). The API calls are invoked by DFTB+ at appropriate stages—either when the matrix needs to be initialized or when it is ready for reading. Matrices can be passed in a distributed form with BLACS descriptors, or using LAPACK triangular storage. The ASI API enables integration of DFTB+ in various multiscale simulations.^152^ and in electronic structure machine-learning workflows.^153^

Reference 152 also describes API bindings for exchange of sparse data including the Hamiltonian, overlap, and density matrices. These bindings define local clusters around atoms and bonds, so are largely boundary-condition agnostic, being suitable for geometries covering cluster, periodic, open-boundary transport, etc.

## 16 Software Engineering Improvements

### 16.1 Modern Fortran MPI and ScaLAPACK Wrappers

In order to enable efficient calculations on large systems, DFTB+ employs distributed-memory parallelization. For standard numerical operations—such as matrix–matrix multiplication or solution of eigenvalue problems—it uses external parallel libraries, including ScaLAPACK,^154^ ELSI,^155^ and PARPACK.^156^ Additionally, DFTB+ specific parallel algorithms are implemented using the stand-alone MpiFx library^157^ which offers robust high level Fortran interfaces to the Message Passing Interface (MPI)^89^ framework. As a recent development, shared objects, which must be accessible in all processes on a node, can now be stored once and accessed by all processes on that node without replication, realized through “shared memory windows” that were added to the MPI standard.^89^ The functionality is accessed via the according high-level interfaces in MpiFx.

A similar initiative, ScaLapackFx,^158^ was launched to wrap the Fortran 77-style ScaLAPACK calls. Developed primarily by the DFTB+ community, ScaLapackFx is also available as a stand-alone open source library.

### 16.2 Unit Testing via the Fortuno Unit Testing System

To ensure software reliability, rigorous testing is a fundamental aspect of the DFTB+ development process. DFTB+ has employed regression testing from the very beginning, where output generated from specific input data is compared against stored reference values. This approach helps to automatically detect unintended modifications in the program’s behavior, preventing programming errors that could compromise existing functionality. To further enhance this process, unit testing can be introduced alongside regression testing. Unit tests evaluate individual code components—typically functions—in isolation, allowing for more granular verification of correctness. Recently, we have begun implementing unit tests for various parts of the core DFTB+ code as well as the MpiFx library. We utilize the Fortuno unit testing framework,^159^ which supports unit testing for serial, MPI- and coarray-parallelized code. Fortuno reduces the amount of boilerplate code required for writing tests, enabling simple unit tests to be implemented as subroutines without arguments. Additionally, the use of the Fypp preprocessor^160^—already integrated into DFTB+ for conditional compilation and automatic generation of interface variations—allows for automated test registration. This automation enhances usability and provides a Fortran testing framework in line with those available in other programming languages.

## 17 Conclusion

We have reviewed recent developments that have significantly expanded the capabilities of DFTB+, including extensions to hybrid functionals, multipolar electrostatics, constrained electronic states, excited-state methods, and real-time electron dynamics. These innovations, together with advances in software engineering and parallelization, have enabled accurate, large-scale quantum simulations across diverse applications in chemistry, materials science, and biophysics with our software package.

While these achievements have broadened the scope of DFTB+, some inherent limitations still remain, including the reliance on minimal basis sets, two-center approximations, and empirical parametrizations, which can restrict accuracy for strongly correlated or highly polarizable systems. Despite progress, systematic parameter generation and their universal transferability continue to be important challenges.

Future directions include the development of broader, automated parametrization workflows, improved treatment of many-body effects, and enhanced hybrid CPU-GPU-accelerated scalability to support simulations of increasingly large and complex systems. Additionally, we are exploring various ways of coupling DFTB+ to machine learning frameworks, in order to ease the creation and to improve the efficiency of hybrid models, where parts of the DFTB or xTB models are enhanced with machine learned predictions. Advances in all these areas will be crucial for pushing the accuracy-efficiency frontier of DFT-based tight binding methods. Overall, DFTB+ stands as a flexible, community-driven platform bridging fast atomistic simulations with quantum mechanical fidelity, and continued innovation promises to further extend its reach across materials science, chemistry, condensed mater and biophysics.

## Supplementary Material



## Notes

Geometries of the structures used to produce the data for Figures 2–4, 6–10 in a ZIP archive (geometries.zip) (ZIP)
